# Accuracy and Speed of Emotion Recognition With Face Masks

**DOI:** 10.5964/ejop.11789

**Published:** 2024-02-29

**Authors:** Arben Hysenaj, Mariel Leclère, Bernard Tahirbegolli, Dorentina Kuqi, Albane Isufi, Lulejete Prekazi, Nevzat Shemsedini, Driton Maljichi, Rina Meha

**Affiliations:** 1 Faculty of Social and Psychological Sciences, Heimerer College, Prishtina, Kosovo; 2 Institute for Sociological, Political and Juridical Research, University of Ss. Cyril and Methodius, Skopje, North Macedonia; 3 Department of Clinical Psychology and Health Psychology, University of Bamberg, Germany; 4 Institute of Sports and Preventive Medicine, Saarland University, Saarbrücken, Germany; 5 Department of Cognitive Psychology, Aix-Marseille University, Marseille, France; 6 National Sports Medicine Center, Prishtine, Kosovo; The Maria Grzegorzewska University, Warsaw, Poland

**Keywords:** emotions, face mask, accuracy, speed, emotion recognition, COVID-19

## Abstract

Wearing face masks is one of the important actions to prevent the spread of COVID-19 among people around the world. Nevertheless, social interaction is limited via masks, and this impacts the accuracy and speed of emotional perception. In the present study, we assess the impact of mask-wearing on the accuracy and speed of emotion recognition. Fifty people (female n = 39, male n = 11) aged 19–28 participated in the study (M = 21.1 years). We used frontal photos of a Kosova woman who belonged to the same participants’ age group, with a grey background. Twelve different pictures were used that showed the emotional states of fear, joy, sadness, anger, neutrality, and disgust, in masked and unmasked conditions. The experiment was conducted in a controlled laboratory setting. Participants were faster for identifying emotions like joy (1.507 ms) and neutral (1.971 ms). The participants were more accurate (emotions identification) in unmasked faces (M = 85.7%) than in masked faces (M = 73.8%), F(1,98) = 20.73, MSE = 1027.66, p ≤ .001, partial η² = 0.17. Masks make confusion and reduce the accuracy and speediness of emotional detection. This may have a notable impact on social interactions among peoples.

The global spread of coronavirus disease 2019 (COVID-19), resulting in the World Health Organization (WHO) declaring COVID-19 as a pandemic, has become a medical serious concern across more than 200 countries worldwide (Wang et al., 2020; World Health Organization [WHO], 2020). To restrain the spread of the virus, by mid-2020 in addition to restrictions in traveling, in-house isolation or quarantine, face masks have become a pervasive feature in the everyday lives of many citizens as governmental authorities made their use compulsory in many circumstances (Chopra et al., 2020; Marini et al., 2021).

In addition to positive medical impact in terms of preventing the virus from spreading to those who are most vulnerable (Wu & McGoogan, 2020), face masks, per definition, cover a major part of the human face, which can crucially affect social interaction. In recent years, studies provide evidence of the impact of face masks on emotion recognition. By making the mouth invisible, emotional expressions are more difficult to interpret (Calbi et al., 2021; Noyes et al., 2021) due to compromising of the facial mimicry and behavioral synchronicity (Palagi et al., 2020). Face masks influence the human ability to infer emotions by observing facial configurations (Gori et al., 2021), to perform perceptual face matching tasks (Carragher & Hancock, 2020), decreasing confidence in expression identification and perception of intensity for all expressions (Pazhoohi et al., 2021). Consequently, covering face impede the ability to perceive socially relevant information of great importance in our everyday life (Marini et al., 2021). Given that different parts of the face are informative about the different emotions a person experiences (Bassili, 1979), the lack of salient stimuli might impact the ability to retain and consolidate learning and memory phenomena underlying face recognition (Ferrari et al., 2021), which significantly effects on daily activities and social interactions (Freud et al., 2020).

While understanding emotions is crucial for social interaction, recent findings suggest that emotion readings were better in the part around the mouth than in fixing the gaze in the eye (Blais et al., 2017; Marini et al., 2021). Researchers studying which parts of the face are associated with specific emotion recognition; show that the part around the mouth is more important in identifying positive emotions like happiness, and the eyes in identifying emotions of aggression, fear and despair (Bombari et al., 2013; Kim et al., 2022), while for neutral emotions, the focus is on both the eyes and the mouth face region (Eisenbarth & Alpers, 2011). Recent studies (Grenville & Dwyer, 2022; Nestor et al., 2020) investigating the implications of mask-wearing on inferring emotions from facial configurations shows that face masks impair mainly positive social interactions, hindering the perception of positive emotions and increasing the perception of negative emotions. On the other hand, a study done by Schurgin et al. (2014) shows that the focus of difference for positive emotions is mainly the part of the lips while in the emotions of sadness the focus was on the eyes. It is also easier for children to recognize the emotion of sadness through the eyes, but not others (Guarnera et al., 2015). Some studies suggest that mask-wearing may make it more challenging to recognize positive emotions while potentially affecting the perception of negative emotions (Spitzer, 2020). These changes can have critical implications for various aspects of social interaction, including perceptions of trustworthiness, likability, and closeness (Grundmann et al., 2021), as well as potential influences on behavior change (Nestor et al., 2020).

In addition, the mask also affects the speed of emotion recognition. The study of Fox et al. (2000), investigating whether speed and spatial cues affect an observer's judgments of emotion showed that participants were likely to produce angry and happy expressions more quickly, in contrary to sad expressions, identified more slowly. Although this study does not specifically address the pandemic or face masks, it provides valuable insights into the rapid detection of facial expressions, especially those conveying anger or threat.

In the light of the ongoing COVID-19 pandemic in 2021, we seized a unique opportunity to investigate how the reading of facial emotions may change when individuals are required to interact with others wearing face masks. The widespread use of face masks as a preventive measure during this period presented a compelling societal backdrop for our research. We hypothesize that the use of face masks during the COVID-19 pandemic has a significant impact on the accuracy of emotion recognition. Specifically, we anticipate that the masking of facial features, particularly the mouth area, may lead to reduced accuracy in perceiving emotions. Additionally, we hypothesize that the speed of emotion recognition is affected by face mask-wearing. We expect that participants may experience delays in identifying emotions when crucial facial expressions are partially obscured by masks.

## Method

### Participants

Fifty people (Female *n* = 39, Male *n* = 11) aged 19–28 years (*M* = 21.1 years; *SD* = 2.2) participated in the study. Participants were recruited from the Prishtina region in Kosovo, they were students of medical sciences from a private higher institution. Prior to enrollment all participants gave written permission and completed a questionnaire before performing the emotion recognition task. This questionnaire consisted of ten questions regarding the participants’ sociodemographic characteristics. The sample size (50 participants) showed 0.93 power (1-β) when analyzed with the G-power program for the difference between two dependent means, for two tails, α = 0.05, and effect size 0.5.

### Materials

**Figure 1:**
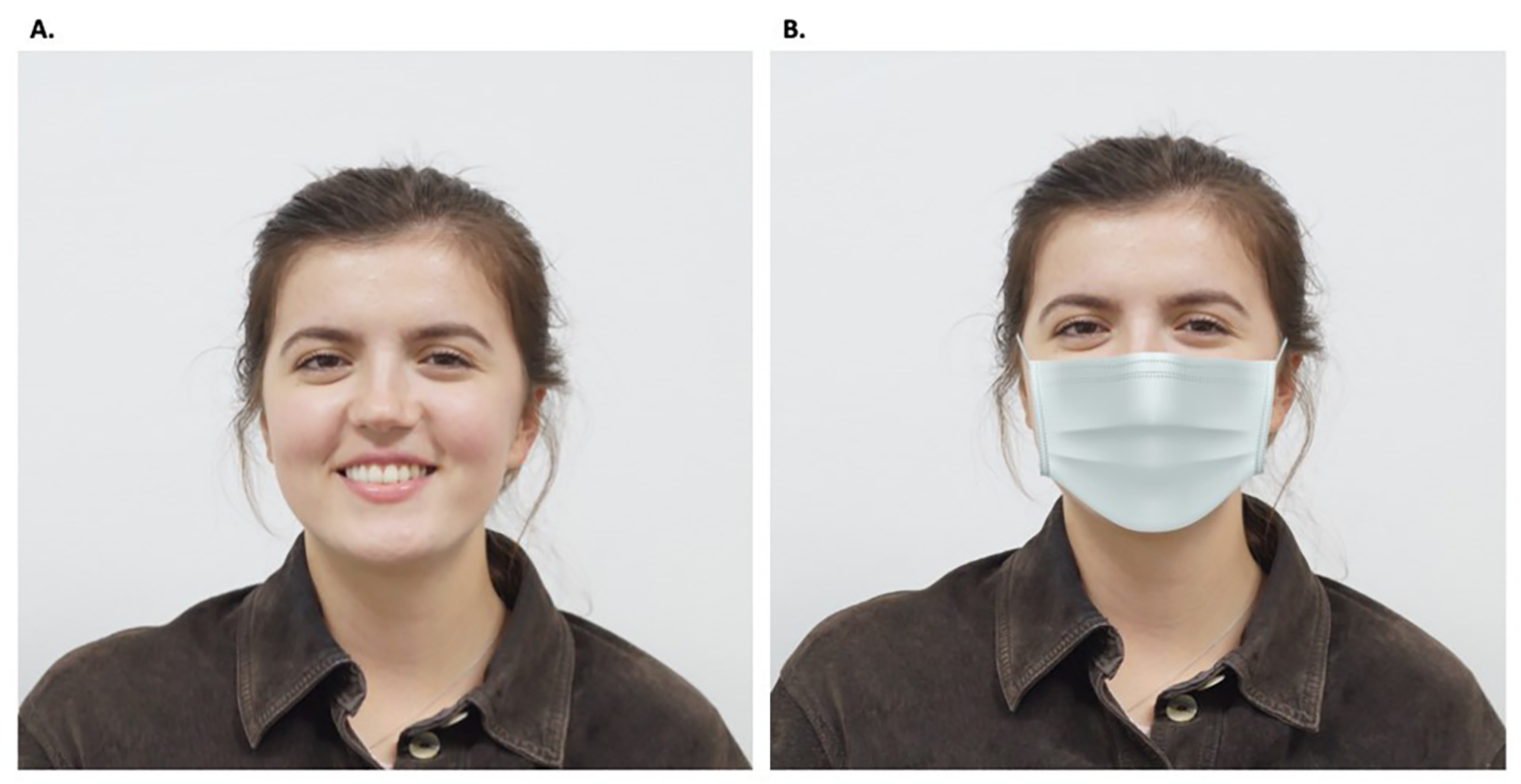
The Model Showing One of the Six Different Emotions (i.e., Joy) Without a Mask (A.) and Wearing a Mask (B.)

For all face stimuli, we used frontal photos of Kosova woman who belonged to the same participants’ age group, with a grey background. Twelve different pictures were used that showed the emotional states of fear, joy, sad, angry, neutral, and disgust, in masked and unmasked condition. We drew a conventional (grey) community mask to apply the mask to our six original photographs. The mask drawing was cut out via Photoshop and applied to the several face versions individually. Afterwards, realistic shadows were applied to make the model wearing the face mask look as natural as possible (Figure 1). A pre-test was conducted, revealing that emotions were correctly identified when no mask was worn.

### Procedure and Design

The experiment has been run on a Windows 10 Version 21H1 and was conducted between June and July 2021. Participants were individually tested in one session that lasted approximately 10–15 minutes. Before encountering the experimental task, participants were told to perform a demonstration about how the trials would be presented. Participants practiced this demonstration task on six random trials (three masked and three unmasked trials) to get familiarized with the procedure of the experiment. The order of the stimuli was randomized across participants, and each participant was exposed to the complete set of stimuli one by one. Participants were asked to assess the depicted person's emotional state spontaneously using a list of six emotions (fear, joy, sadness, anger, neutral and disgust) shown by the different versions of the condition (masked - unmasked). There was no time limit for giving a response. However, as soon as each participant thought they had recognized the emotion, they had to answer as fast as possible. The experimental stimuli (young woman picture with face masked or unmasked) were presented in the center of a 17-inch computer by Lenovo c360 PC 10147. The experiment was controlled by PsychoPy software. The program generated the displays and recorded latencies to the nearest millisecond. Each trial began with a blank screen for 500 milliseconds (ms), followed by a signal (‘+’) presented for 500 ms in the center of the screen. Then, the picture was displayed on the computer screen and was presented until the participant gave his/her response. Participants were asked to press on one of the six letter keys (‘A’, ‘E’, ‘F’, ‘H’, ‘I’, ‘L’) of the QWERTZ keyboard. Letter keys have been decided according to the balance between the use of each hand. Thus, the ‘A’, ‘E’, and ‘F’ keys were assigned to the left hand and ‘H’, ‘I’, ‘L’ were assigned to the right hand. Additionally, the three first letters of each emotion were sticked to each response key. After each picture, a blank screen for 500 ms was finally displayed at the end of each picture and before the next picture appeared.

The results are reported in two main sections. First, we analyze the differences between condition faces and emotion states with accuracy percentages. Then we examine the variations in latencies (in milliseconds) between our dependent variable (emotion) and our factor (masked vs. unmasked face). Unless otherwise stated, all differences are significant at least at *p* < .05, and effect sizes were computed as partial eta-squared ($\eta_{p}^{2}$) for ANOVAs. All analyses were performed through Statistica 12 software. The procedure of this study complied fully with the provision of the Helsinki Declaration regarding research on human participants.

## Results

### Emotion Recognition Accuracy

If participants recognized the right emotion on the photos, the emotion recognition accuracy was coded 1; otherwise, 0. ANOVAs were performed on mean percentage of accuracy with 2 (Condition face: Masked, Unmasked) x 6 (Emotion: Fear, Joy, Sadness, Anger, Neutral, and Disgust) design with repeated measures on the last factor.

Participants were more accurate when they had to identify facial emotions in faces unmasked (*M* = 85.7% ± 1.8) than in faces masked (*M* = 73.8% ± 1.8), *F*(1,98) = 20.73, *MSE* = 1027.66, *p* ≤ 0.001, $\eta_{p}^{2}$ = 0.175. Moreover, as shown by the data for each emotional state (see Table 1), participants were more accurate for identifying the positive emotion of joy (*M* = 98.5% ± 0.7) and neutral emotion (*M* = 91.3% ± 1.9), than for identifying other emotions, connoted as negative emotions, like fear (*M* = 86.0% ± 1.9), anger (*M* = 82.0% ± 2.6), sadness (*M* = 62% ± 3.1), and disgust (*M* = 58.5% ± 3.7), *F*(5,490) = 46.07, *MSE* = 561.99, *p* ≤ .001, $\eta_{p}^{2}$ = 0.086. The two-way interaction involving the condition face factor came out significant. The Condition face x Emotion state, interaction *F*(5,490)= 18.85, *MSE* = 561.99, *p* ≤ .001, $\eta_{p}^{2}$ = .037, revealed that the difference in the accuracy for identifying emotions between masked and unmasked faces was bigger with sad state, 52%, *F*(1,98) = 68.47, *MSE* = 987.24, *p* ≤ .001, $\eta_{p}^{2}$ = .411, than with disgust state, 15.0%, *F*(1,98) = 4.17, *MSE* = 1348.47, *p* = .043, $\eta_{p}^{2}$ = .041. No other significant effects came out when we compared masked and unmasked faces with fear, joy, anger, neither neutral states (*F* < 1).

**Table 1:** Mean Correct Emotional Recognition and Mean Solution Latencies in Each Condition Face as a Function of Each Emotion State

Condition Face	Emotion State						Mean
	Fear	Joy	Sadness	Anger	Neutral	Disgust	
Accuracy (in %)							
Masked	83.5 ± 2.7	97.5 ± 0.9	36.0 ± 4.4	81.5 ± 3.7	93.0 ± 2.8	51.0 ± 5.2	73.8 ± 1.8
Unmasked	88.5 ± 2.7	99.5 ± 0.9	88.0 ± 4.4	82.5 ± 3.7	89.5 ± 2.8	66.0 ± 5.2	85.7 ± 1.8
Total	86.0 ± 1.9	98.5 ± 0.7	62.0 ± 3.1	82.0 ± 2.6	91.3 ± 1.9	58.5 ± 3.7	79.7
Differences	5.0 n.s	2.0 n.s	52.0*	1.0 n.s	3.5 n.s	15.0*	11.9*
Solution Latencies (in ms)							
Masked	2.804 ± 0.2	1.632 ± 0.1	3.129 ± 0.1	2.449 ± 0.1	1.988 ± 0.1	2.694 ± 0.1	2.449 ± 0.7
Unmasked	2.829 ± 0.2	1.382 ± 0.1	2.445 ± 0.1	2.519 ± 0.1	1.954 ± 0.1	2.768 ± 0.1	2.316 ± 0.7
Total	2.816 ± 0.1	1.507 ± 0.1	2.787 ± 0.1	2.484 ± 0.1	1.971 ± 0.1	2.731 ± 0.1	2.382
Differences	.02 n.s	250*	684*	70 n.s	34 n.s	74 n.s	133 n.s

*p < .05. n.s = non-significant.

### Emotion Recognition Performance

Means of correct solution latencies were analyzed with mixed-design ANOVAs, 2 (Condition: Masked, Unmasked faces) x 6 (Emotion: Fear, Joy, Sadness, Anger, Neutral, and Disgust), with repeated measures on the last factor (Table 1).

The main effect of the condition was not significant (*F* > 2). However, the main effect of emotion was significant, *F*(5,490) = 40.04, *MSE* = 0,71, *p* ≤ .001, $\eta_{p}^{2}$ = .076, and revealed that participants were faster for identifying emotion like joy (1.507 ms ± 0.1) and neutral (1.971 ms ± 0.1) than the other emotions like anger (2.484 ms ± 0.1), disgust (2.731 ms ± 0.1), sadness (2.787 ms ± 0.1) and fear (2.816 ms ± 0.1). The two-way interaction came out significant, *F*(5,490) = 3.07, *MSE* = 0,71, *p* ≤ .01, $\eta_{p}^{2}$ = .006. It revealed that the difference in the speed to identifying emotions between masked and unmasked faces was bigger with sadness state, 684 ms, *F*(1,98) = 23.82, *MSE* = 0,49, *p* ≤ .001, $\eta_{p}^{2}$ = .196, than with joy state, 250 ms, *F*(1,98) = 5.87, *MSE* = 0,26, *p* = .017, $\eta_{p}^{2}$ = .057, with a faster identification in unmasked than masked faces. No other significant effects came out when we compared masked and unmasked faces for fear, anger, neutral neither disgust state (*F* < 1). However, interestingly, the latencies’ appeared faster, in anger, fear and disgust states, in masked than in unmasked faces.

## Discussion

In the present study, we investigated the impact of face masks on emotion recognition, which has garnered significant attention during the COVID-19 pandemic, as it holds substantial consequences for daily social interactions, particularly in emotion identification. Our first hypothesis posited that the use of face masks during the pandemic would significantly impact the accuracy of emotion recognition. Specifically, we anticipated that masking facial features, especially the mouth area, could lead to reduced accuracy in perceiving emotions. In this regard our results align with existing data (Bani et al., 2021) and confirm that emotion recognition is indeed more challenging when faces are masked. However, it is essential to note that this reduction in recognition accuracy was more pronounced for certain emotions, with joyful and neutral expressions being exceptions. This observation partially aligns with previous research that utilized various forms of occlusions (e.g., cardboard enclosures, Bubbles technique), including face masks (Carbon, 2020). Our findings are also consistent with reports of face masks having a negligible effect on the recognition of neutral expressions (Noyes et al., 2021). This lack of effect on neutral stimuli may be attributed to previous research emphasizing the importance of the eye region in the expression and recognition of neutrality (Blais et al., 2012; Wegrzyn et al., 2017), as closing the mouth region with a mask may not significantly affect such recognition. Notably, our results showed increased accuracy in recognizing joyful expressions in the mask condition, which aligns with Pazhoohi et al.'s (2021) findings of greater confidence in identifying happiness and sadness in masked faces of women.

Moreover, our second hypothesis proposed that the speed of emotion recognition would be affected by face mask-wearing. We expected that participants might experience delays in identifying emotions when crucial facial expressions were partially obscured by masks. In this regard, our analyses on the reaction times for recognizing emotions in both masked and unmasked conditions revealed that there was no significant main effect between the conditions. However, participants were notably faster at identifying joyful and neutral emotions. These findings are consistent with previous research indicating that happiness is the fastest emotion to be recognized (Martinez & Du, 2010). In line with this evidence, we observed that sadness and anger constitute the group of emotions that require the longest time for recognition, approximately ten times slower than happiness (Martinez & Du, 2010). Interestingly, the neutral emotion recognition time differed, with evidence suggesting it takes three to four times longer to recognize than happiness (Martinez & Du, 2010).

In this study, we employed a singular gender of mannequin to express emotions, prompting a discussion within the broader context of gender implications in emotion recognition. Previous research has explored the connections between specific emotions and gender bias, suggesting that certain expressions may be more strongly associated with either men or women (Hess et al., 2009). It is crucial to note, however, that conflicting findings exist, as recent research indicates no significant primary influence of model gender on the perception of emotions (Rinck et al., 2022). Aligned with this recent perspective, our current investigation suggests that participants' emotion recognition skills may not have been affected by the gender of the selected model. To enhance our understanding of these dynamics, future research should adopt a more comprehensive, gender-diverse approach, incorporating stimuli representing both male and female genders.

This study highlights the challenges posed by face masks in interpreting emotions solely through facial expressions. Nevertheless, these challenges should not be seen as a reason to avoid wearing masks when required. Other cues, such as voice characteristics (Golan et al., 2006), body posture (Aviezer et al., 2008), head orientation (Sauer et al., 2014), and social context aspects (Mondloch, 2012), provide additional information for emotional recognition. Moreover, direct verbal communication remains a valuable tool for understanding a person's mental state.

### Limitations

There are a few limitations in this study. The first limitation is that just one gender was used as a mannequin to display emotions. The researchers made this decision because they wanted to keep the testing duration short and maintain uniformity with one person. Another limitation is that the researchers did not reveal the entire body/posture position so that other perceptions associated with the masked face could be elicited. The use of a limited number of participants may have constrained our ability to detect smaller effects and generalizability to a broader population. Our study included medical students, and it is important to acknowledge that this specific demographic might not fully represent the general population. The gender-based study sample was too small to perform specific statistical analysis and assumptions. Additionally, our study was conducted during the mid-pandemic period, and the societal context was characterized by evolving public health measures and individual behaviors.

### Conclusion

In conclusion, we can say that this study also revealed that the COVID-19 pandemic, specifically the use of face masks to prevent the spread of infection, has provided valuable insights into the challenges associated with recognizing emotions when individuals are wearing face masks. We observed that face masks indeed have a significant impact on emotion recognition, making it more difficult for individuals to perceive emotions displayed on masked faces, particularly for certain emotions. Leaving room for misunderstandings in nonverbal communication and necessitating verbal communication.

## Human and Animals Rights

No animals were used for studies that are the basis of this research. All the human procedures used were in accordance with the ethical standards of the committee responsible for human experimentation (institutional and national), and with the Helsinki Declaration.
